# No Evidence of Association between *Toxoplasma gondii* Infection and Financial Risk Taking in Females

**DOI:** 10.1371/journal.pone.0136716

**Published:** 2015-09-24

**Authors:** Lasha Lanchava, Kyle Carlson, Blanka Šebánková, Jaroslav Flegr, Gideon Nave

**Affiliations:** 1 Center for Economic Research and Graduate Education and Economics Institute (CERGE-EI), Prague, Czech Republic; 2 Department of Humanities and Social Sciences, California Institute of Technology, Pasadena, United States of America; 3 Department of Biology, Faculty of Science, Charles University in Prague, Prague, Czech Republic; 4 Department of Computation & Neural Systems, California Institute of Technology, Pasadena, United States of America; Middlesex University London, UNITED KINGDOM

## Abstract

**Background:**

Past research linked *Toxoplasma gondii* (TG) infection in humans with neurological and mental disorders (e.g., schizophrenia, Alzheimer’s disease and attention disorders), irregularities of the dopaminergic and testosterone system, and increased likelihood of being involved in traffic accidents.

**Methodology/Principal Findings:**

We test for an association between TG infection and financial decision-making (DM) using a case-control design in a sample of female Czech students (n = 79). We estimate each subject's risk attitude and loss aversion using an experimental economic task involving real monetary incentives. We find no significant evidence that either measure of decision-making is associated with TG infection.

**Conclusion:**

We were unable to find evidence of an association between TG infection and financial decision-making in females.

## Introduction

Accumulating evidence from the lab and field show that human decision-makers display a great level of individual heterogeneity in economic preferences [1–6]. Although the diversity of preferences is a well-documented phenomenon at the descriptive level, its roots are poorly understood. Accordingly, researchers seek to understand the origin of the observed variability and whether cultural, educational or biological factors underlie the heterogeneity of economic preferences [5–11]. In recent years, scholars have suggested that interplay of genetics and cultural influences is responsible for shaping personality traits and other economic preferences [12–15]. Furthermore, biological factors such as circulating levels of the steroid hormones cortisol [16] and testosterone [17–19], and individual differences in the functionality of neurotransmitter systems, e.g. dopamine and serotonin [20–22] have been found to modulate decision-making (DM) under uncertainty.

A biological factor that might partially explain the heterogeneity of economic preferences and has so far been overlooked by researchers is the composition of parasitic microorganisms residing within the human body [23]. Evidence shows that parasites can cause significant behavioral changes in various species, from insects to mammals [24–31], where a well-known ethological theory, the ‘behavioral manipulation hypothesis’ postulates that parasites specifically manipulate host behaviors so as to increase the fitness of the parasite [29,32]. For example, the parasite *Toxoplasma gondii* (TG), which is the subject of this study, must be transmitted from an intermediate host to the definite host, a feline predator, in order to sexually reproduce. Therefore, TG would increase its fitness by manipulating the intermediate hosts’ behavior so as to increase contact with felids.

We focus on TG for several reasons. First, TG has been shown to manipulate the behavior of some of its mammalian hosts such that the probability that a feline predator will capture them increases; for example, TG influences rodents’ vigilance and their ability to recognize novel stimuli, causes prolonged reaction times and even turns rodents’ innate fear of the cat odor into an attraction [33–36]. While it is true that modern humans are rarely eaten by cats and therefore it is unlikely that TG-related changes in human behavior have an impact on predation risk, it is plausible that TG targets human brain tissues as a byproduct of its evolution in rodents. Humans’ immune responses to the latent infection may also induce behavioral changes. Furthermore, since in early stages of evolution apes constituted a significant share of the prey of feline predators, TG’s act on human behavior could also be an evolutionary remnant [37]. Finally, an estimated 30% of humans worldwide have been infected by TG, but this rate varies substantially between countries, making the parasite a candidate for generating behavioral variation between populations.

Although latent TG infection has been considered harmless for many years following its discovery (While latent infection was generally considered to be harmless, it is well-recognized that the acute TG infection can be harmful, especially for immunocompromised or fetuses [38]), studies over the past few decades have associated it with various psychiatric and neurological problems, including schizophrenia, Alzheimer’s disease, and attention disorders [37,39–41]. Many of the behavioral effects of TG in rodents have been extended to humans. Thus, TG infected humans show prolonged response times in cognitive tasks and TG infection is suspected to underlie individual differences in various personality traits [26,42]. Furthermore, several independent studies associate TG infection with increased rates of traffic accidents, which might be mediated by impaired decision-making capabilities or changes in sensitivity to risk and losses [43–45].

TG infection can cause functional changes in the dopaminergic system [37,46–48], which plays a key role in DM and is particularly involved in shaping one’s attitudes towards risk and loss [20,22,49,50]. Moreover, the genome of TG contains two genes for the enzyme tyrosine hydroxylase, which is essential for dopamine synthesis [51]. Finally, TG is also associated with gender-specific individual differences in the circulating levels of the hormones testosterone and cortisol [52,53], which are correlated with individual differences in risk taking in economic DM [17,54]. Although causality has not been established in humans (due to methodological and ethical limitations) experiments on rodents shows that TG causes changes in testosterone and not vice-versa [55].

Given the growing evidence of behavioral and physiological effects of TG infection in humans, we hypothesized that TG-infection would have an influence on economic DM. The current case-controlled study used a well-established experimental task [56,57] to investigate individual differences in two components of financial DM, namely risk and loss aversion. Risk aversion is the tendency to prefer a guaranteed payoff to a gamble with equivalent expected value [58,59]. By and large, people are willing to pay a premium in order to reduce the variance of their choice outcomes, even when the riskier option has a greater expected value. Loss aversion is human’s tendency to overweight losses relative to gains [60,61], a phenomenon that has been repeatedly documented in lab experiments using both money and goods [62–64] and in field data [65–68]. A large body of literature reports that biological factors such as gender, genetic variability and physiological state (such as hormone levels) underlie individual differences in people’s attitudes toward risk and loss [17,54,56,69–71]. Furthermore, loss aversion and risk aversion exist across species [72,73], making them candidate descriptive measures for decision parameters that might be affected by biological factors.

## Materials and Methods

### Subjects

Seventy-nine female subjects (mean age, 23.89; S.D. 3.65) participated in the experiment. Participants were recruited from the pool of current and former biology students of the Faculty of Science, Charles University, Prague. All subjects were previously tested for toxoplasmosis and rhesus (RhD) status (see detailed methods below). All participants provided a written informed consent. Subjects’ recruitment and data handling were performed in compliance with the Czech legislation in force and were approved by the Institutional Review Boards of the Faculty of Science, Charles University and the Department of Humanities and Social Science, Caltech. The student volunteers often take part in many studies, most of which are not related to TG infection. Crucially, TG was not mentioned in the study recruitment invitation, and the experiments were run at the University of Economics—not the usual location for TG-related experiments. Therefore, it is unlikely that subjects suspected that the experiment was related to TG research. Moreover, no data or hypotheses concerning possible effects of TG on the risk-taking behavior had been published before the study started.

### Experimental Procedures

The experimental sessions took place in November and December 2013 in the Laboratory of Experimental Economics (LEE) in Prague. Upon arrival, participants were randomly assigned to isolated computer stations where they could not interact with each other. Subjects received a printed copy of the instructions (see supporting information (SI)). After reading the instructions, subjects were asked to complete a quiz to ensure their understanding of the task. The experimenter (same person in all sessions) was responsible for verifying the correctness of their answers and in case of a wrong answer provided an explanation, until subjects fully understood the task. After all subjects had answered the quiz questions correctly, they proceeded to the task. The task was designed to explore subjects’ attitudes towards monetary risk and potential losses and was programmed in z-Tree [74] in English. After the completion of the task and an additional, unrelated task, participants filled out a questionnaire about their socio-economic background, health, personality, and general attitude towards risk. The experimental sessions lasted approximately 90 minutes and on average subjects earned 350 Czech korunas (CZK), equivalent to 17.5 USD according to the exchange rate at that time. After making their decisions, subjects were privately paid in cash.

### Financial decision-making task

**Fig 1:**
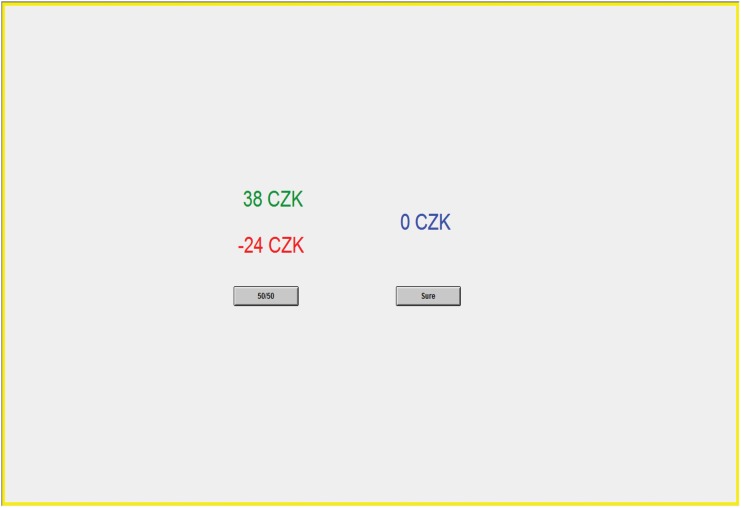
Risk task: A sample screenshot from the study. The two numbers on the left represent the gamble’s possible gain and loss amounts (*Top* and *Bottom*, respectively). The number on the right represents the guaranteed amount. Participants had to indicate which option they wanted to choose by clicking the corresponding button.

Subjects were endowed with 250 CZK and made a series of 140 forced choices between different pairs of monetary gambles (S3 Table in SI lists the entire set of pay-offs used). Each pair contained a sure option (SO) which paid S CZK for sure, and a risky option (RO), which delivered a gain of G CZK and a loss L CZK each with an equal probability of 50% (Fig 1). The task was used to measure risk attitudes and loss aversion in previous studies [56,57]. Choices were presented in four pseudo-randomly ordered blocks of 35 decisions, such that block order was counterbalanced across participants. Gamble positions (left or right) and gamble outcomes were randomized across participant. Subjects received no immediate feedback about the lottery outcomes and were informed that at the end of the experiment, one trial would be selected at random and the payoff associated with the selected option would be implemented. The payoffs and initial endowment of 250 CZK were such that no subject could end with negative earnings.

### Immunological Tests for Toxoplasmosis

Serological tests took place between 2006 and 2013 in the National Reference Diagnostic Laboratory for Toxoplasmosis, National Institute of Public Health, Prague. Specific IgG and IgM antibody titres were determined by ELISA (IgG: SEVAC, Prague, IgM: TestLine, Brno), optimized for early detection of acute toxoplasmosis [75] and by complement fixation tests (CFT, SEVAC, Prague) which are more sensitive and therefore more suitable for the detection of latent TG infection [76]. The titre of anti-TG antibodies in sera was measured in dilutions between 1:8 and 1:1024. The subjects with negative results of IgM ELISA (positivity index<0.9) and both CFT titres higher than 1:8 and IgG ELISA>250 optical units, i.e. approximately 10 IU/ml, were considered latent toxoplasmosis positive. Individuals with ambiguous diagnosis, e.g., different result of CFT and ELISA, were excluded from the study.

Six of the non-infected subjects were tested prior to 2010; nine subjects were tested between April 2010 and November 2010; seven subjects were screened from October 2011 to November 2011. The remaining 18 non-infected subjects were tested between March 2012 and April 2013. The seroprevalence of TG increases from 8% in women aged 12 to 19 to 15% in women aged 20 to 29 [77]; these numbers are comparable to the Czech Republic population [78]. As the age range of women in our study was 20 to 26 (mean 23.15; SD 1.64), and given that the non-TG women tested negative in prior years (12 to 19 years of age), it is safe to deduce that 7% is an upper bound for the likelihood that any of the control group subjects had contracted the parasite between the time of the serological examination and the behavioral task. All of the analysis replicates when we exclude the 6 subjects that were tested prior 2010 (see S5–S7 Tables in supplementary material section).

### RhD Examination

Several studies over the past years have that RhD phenotype moderates several effects of TG on human personality, reaction times and behavior [79–82]: generally, RhD positive subject are highly resistant to TG related effects [83], where TG has opposite effects on RhD negative homozygotes and RhD positive heterozygotes [84]. Therefore, it is important to include RhD phenotype in the analysis as a covariate.

We used a standard agglutination method for RhD examination. A constant amount of anti-D serum (human monoclonal anti-D reagent; Seraclone, Immucor Gamma Inc.) was added to a drop of blood on white glass plate. Red cells of RhD-positive subjects were agglutinated within 2–5 minutes.

## Results

### Summary Statistics

We present summary statistics in Table 1. The experiment was a case-control study; thus, the prevalence of neither TG-infected nor RhD negative subjects reflected real frequencies in Czech population: According to a recent study [78] the prevalence of latent toxoplasmosis in women of childbearing age in the Czech Republic was 20% as of 2007. The rate of RhD negative subjects in the Caucasian population was about 16% [76]. Among 79 subjects, 39 were TG-infected and 40 were TG-free. There were about twice as many RhD positive subjects (N = 53) as RhD negataive ones (N = 26) and their distribution among TG-infected and TG-free subjects were identical. TG-infected subjects were slightly older (t(77) = -1.87, P<0.07).

**Table 1:** Summary statistics for the sample.

	All			Toxo +			Toxo -			P^a^
Variable	Obs.	Mean	SD	Obs.	Mean	SD	Obs.	Mean	SD	
Age	79	23.89	3.65	39	24.66	4.84	40	23.15	1.64	0.065
RhD positive	53	NA	NA	26	NA	NA	27	NA	NA	NA
RhD negative	26	NA	NA	13	NA	NA	13	NA	NA	NA

^a^ P shows statistical significance for two tailed t test.

### Decision-making task

**Fig 2:**
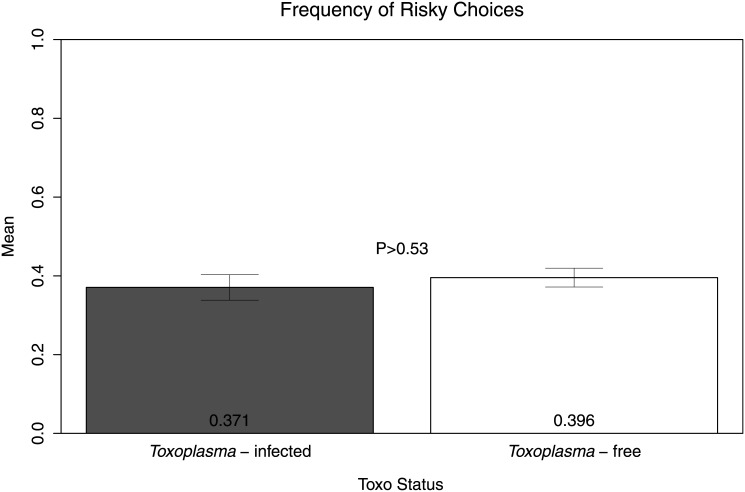
Frequency of risky choices in *Toxoplasma*-infected and *Toxoplasma*-free subjects. The graph shows arithmetic means, standard errors and a p-value.

**Fig 3:**
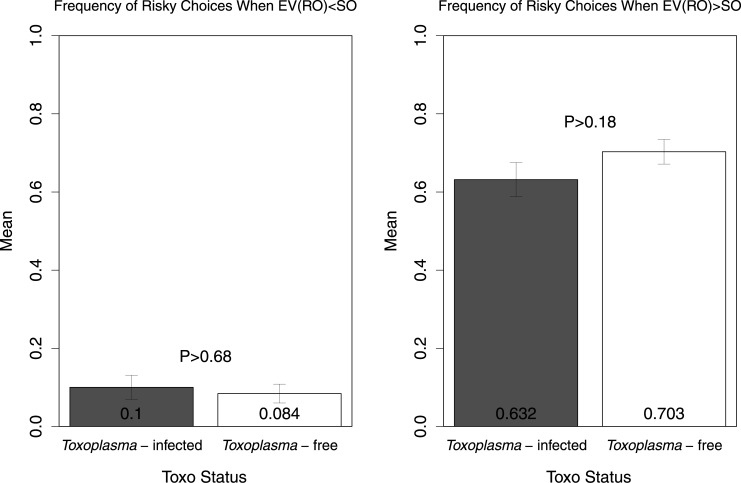
Frequency of risky choices when the expected value of RO is less than SO (left) and the expected value of RO is greater than SO (right) in *Toxoplasma*-infected and *Toxoplasma*-free subjects. The graph shows arithmetic means, standard errors and a p-value.

**Fig 4:**
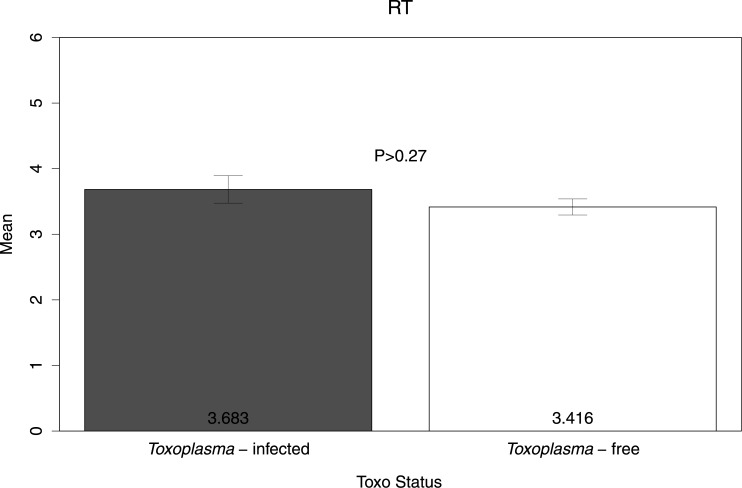
Decision response times (RT) in *Toxoplasma*-infected and *Toxoplasma*-free subjects. The graph shows arithmetic means, standard errors and a p-value.

**Fig 5:**
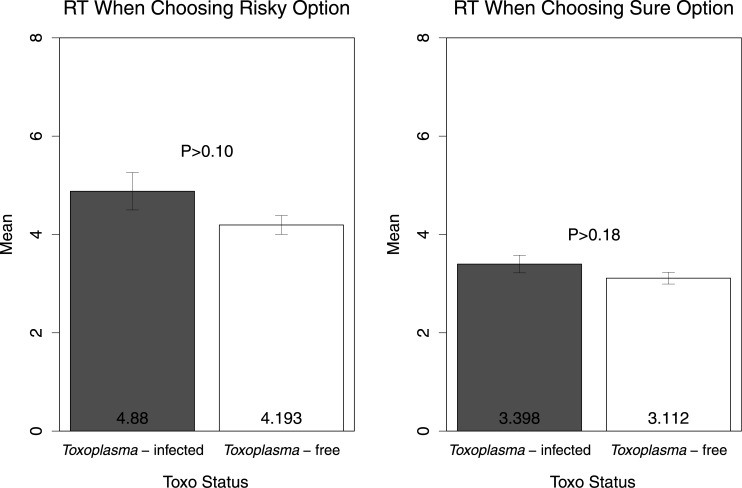
Decision response times (RT) when choosing RO (left) and SO (right) in *Toxoplasma*-infected and *Toxoplasma*-free subjects. The graph shows arithmetic means, standard errors and a p-value.

For each subject, we calculated a raw measure of risk attitude by calculating the frequency of risky choices (i.e., the proportion of trials in which the subject chose the risky option). Fig 2 shows the means of this variable in both TG-infected and TG-free subjects. The difference was not statistically significant (t(77) = 0.61, P>0.53). To further assess risk-seeking behavior, we examined subjects’ choices when the expected value of the gamble was less than the sure option; a two tailed t-test shows that the difference was not statistically significant (t(77) = -0.40, P>0.68, Fig 3, left panel). We also found no significant differences in the frequency of risky choices when the expected value of the gamble was more than the sure option (t(77) = 1.33, P>0.18, Fig 3, right panel). Additionally, we compared average response times (RTs) in seconds (per trial) across the two groups (see Fig 4). In accordance with previous studies reporting slower RTs in TG-infected subjects [42] the TG-infected group average RT was larger than the TG-free counterparts, though the difference was not statistically significant (t(77) = -1.10, P>0.27). We also found no differences in RTs between the two groups when separately examining choices of the risky option (t(77) = -1.62, P>0.10, Fig 5, left panel) and choices of the sure option (t(77) = -1.33, P>0.18, Fig 5, right panel).

We defined the variable *incentive* as the difference between expected value of the gamble and the sure amount and further analyzed the data using logistic regression with clustered standard errors at subject level. The binary outcome variable choice indicated (= 1) if the subject chose the risky option in a given trial. Column (1) in Table 2 reports the results of basic regression. As expected, we found a significant effect of incentive on participants’ decisions (X^2^(1, *N* = 79) = 138.14, P<0.01). Our results show no effect of TG-infection status on subjects’ DM under financial risk (X^2^(1, *N* = 79) = 0.38, P>0.53). In column (2) we added other explanatory variables (age, RhD status) to our basic regression. None of the new explanatory variables were statistically significant, and the magnitude and significance levels of the basic regression coefficients remained virtually unchanged. Next, we investigated the data using a model that also accounts for the variance of the risky option, by adding a variable representing the variance of the risky choice to the original regression. Columns (3) and (4) show the estimation results of the basic regression without interaction terms, with interaction terms, and with additional explanatory variables. Adding the variance did not affect the significance of the coefficients, and the results from columns (1) and (2) are not substantially changed.

**Table 2:** Logistic regression.

	Choice			
Dependent Variable	(1)	(2)	(3)	(4)
Toxoplasma^a^	0.153	0.191	0.154	0.193
	(0.248)	(0.461)	(0.250)	(0.466)
Incentive	0.088***	0.089***	0.088***	0.089***
	(0.007)	(0.007)	(0.008)	(0.008)
Age		0.001		0.001
		(0.031)		(0.032)
RhD^b^		0.095		0.096
		(0.320)		(0.323)
Toxoplasma*RhD		0.516		0.520
		(0.542)		(0.547)
Variance			6.34E-5***	6.36E-5***
			(1.48E-5)	(1.48E-5)
Constant	0.538***	0.569	0.248	0.279
	(0.149)	(0.786)	(0.150)	(0.793)
Restricted Log Likelihood	5377	5359	5303	5285
Observations	11060	11060	11060	11060

Coefficients in all columns are logistic regression estimates, clustered standard errors are in parentheses;

*** indicate significance at 1% level.

^a^ *Toxoplasma* is a dummy variable and equals 1 for *Toxoplasma*-infected subjects.

^b^RhD is a dummy variable and equals 1 for RhD positive subjects.

We analyzed the decision RT data using a mixed-effects linear model. Column (1) in Table 3 reports the results of the basic regression. Across groups, choices of the risky option took considerably longer (z(77) = 10.55, P<0.01). Additionally, when facing higher monetary incentives to take risks, decisions were slower (z(77) = 6.31, P<0.01). There was no difference in decision times between TG-infected and TG-free groups (z(77) = 1.17, P>0.24). In column (2) of Table 3 we repeated the regression by including interaction terms. The Toxoplasma-Choice interaction term was positive but insignificant (z(75) = 1.16, P>0.24). As expected, we also observed a significant negative effect of choice x incentive interaction (z(75) = -10.18, P<0.01), implying that when deciding to take a risk, subjects made the decision faster when the risky option was more attractive. In column (3) in Table 3 we extend our analysis by including additional explanatory variables (age, RhD). None of the new controls have a significant effect on RT.

**Table 3:** Mixed-effects linear regression.

	Response Time		
Dependent Variable	(1)	(2)	(3)
Toxoplasma^a^	0.286	0.210	-0.118
	(0.244)	(0.254)	(0.435)
Incentive	0.010***	0.021***	0.021***
	(0.001)	(0.001)	(0.001)
Choice^b^	0.785***	0.977***	0.977***
	(0.074)	(0.097)	(0.097)
Toxoplasma*Choice		0.140	0.141
		(0.121)	(0.121)
Choice*Incentive		-0.040***	-0.040***
		(0.003)	(0.003)
Age			0.038
			(0.034)
RhD^c^			0.086
			(0.368)
Toxoplasma*RhD			0.408
			(0.523)
Constant	3.125***	3.258***	2.315***
	(0.174)	(0.180)	(0.847)
Log Likelihood	-27469	-27597	-27595
Observations	11060	11060	11060

Coefficients in all columns are logistic regression estimates, clustered standard errors are in parentheses;

*** indicate significance at 1% level.

^a^ *Toxoplasma* is a dummy variable and equals 1 for *Toxoplasma*-infected subjects.

^b^Choice is a dummy variable and equals 1 if subjects chose risky option.

^c^RhD is a dummy variable and equals 1 for RhD positive subjects.

As a final step, we estimated a parametric model inspired by prospect-theory [60] for measuring individual levels of risk aversion (ρ) and loss aversion (λ) using maximum likelihood estimation, in a similar manner to recent studies that investigated individual differences in risk and loss aversion applying the same experimental task [56,57] (see S2 Appendix for detailed method. Table 4 reports summary statistics of the parametric data (S1 and S2 Figs illustrate the model fit). A two-tailed t-test revealed no statistically significant differences in the mean values of ρ and λ between the TG-infected and TG-free groups. We also performed OLS regression analysis with additional explanatory variables and found no significant effect of any explanatory variable on either risk aversion or loss aversion parameters (columns 1 and 2 of Table 5). In addition, we excluded parameter values that were more than two standard deviations away from mean (see S4 Table in SI for individual parameter estimates, nine extreme values were excluded in total). The number of excluded subjects was significantly lower in the uninfected group (2 out of 40) compared to the infected group (7 out of 39, p = 0.04). These subjects were excluded because their risk aversion (ρ) and/or loss aversion (λ) parameters were unusually high or unusually low. The results were robust to excluding these extreme parameter values in the analysis (see S1 and S2 Tables).

**Table 4:** Summary statistics of the parametric data.

	All			Toxo +			Toxo -			P^a^
Variable	Obs.	Mean	SD	Obs.	Mean	SD	Obs.	Mean	SD	
^ρ^	79	0.846	0.145	39	0.829	0.175	40	0.864	0.108	0.29
^λ^	79	1.612	0.892	39	1.762	1.06	40	1.465	0.672	0.139

^a^P shows statistical significance for two tailed t-test.

**Table 5:** Regression analysis of individual parameters.

	^ρ^	^λ^
Dependent Variable	(1)	(2)
*Toxoplasma* ^a^	-0.042	0.269
	(0.056)	(0.356)
Age	-0.001	0.002
	(0.004)	(0.028)
RhD^b^	0.070	0.155
	(0.048)	(0.303)
*Toxoplasma* *RhD	0.017	0.039
	(0.068)	(0.430)
Constant	0.859***	1.311*
	(0.110)	(0.697)
R^2^	0.083	0.036
Observations	79	79

Coefficients in all columns are logistic regression estimates, clustered standard errors are in parentheses;

*** indicate significance at 1% level.

* indicate significance at 10% level.

^a^ *Toxoplasma* is a dummy variable and equals 1 for *Toxoplasma*-infected subjects.

^b^RhD is a dummy variable and equals 1 for RhD positive subjects.

## Discussion

Although latent TG-infection in humans has been considered harmless for many years following its discovery, recent studies suggest effects on human behavior. TG is associated with an increased risk of psychological disorder, changes in personality traits, prolonged response times in cognitive tasks, and a higher likelihood of being involved in a car accident [26, 37,39–45]. TG is also linked to functional changes in the dopaminergic and testosterone systems [51–53], both of which are known to shape one’s attitudes towards risks and losses and partly explain individual differences in economic preferences. In light of these findings and based on the well-established animal literature, the present study is the first to examine a potential link between latent TG infection and financial DM, namely risk and loss aversion, in humans. Using a case-controlled design and a well-established experimental task that was previously used to investigate individual differences in economic DM, we explore two hypotheses relating TG infection to risk-taking and loss aversion. While our study fails to find such a direct link, we acknowledge several limitations to our work.

Our sample is comparable in size to other DM studies that used the same experimental paradigm to explore the influence of psychological and biological factors on financial DM [56,57]. Given the costs of constructing a case-controlled study (e.g. conducting blood-draws and bio-analysis in a large sample of candidate participants), we find this sample size adequate. Yet, as this is the first attempt to test the effects of parasitic infection on human DM, we had no means of conducting detailed power analysis ex-ante. Given the moderate sample size, we cannot rule out very small effects. However, our results are inconsistent with any large effect of TG infection.

Based on recent studies reporting gender-specific effects of TG on several psychological and physiological variables [32,52], the current study used only female subjects. Future studies should further illuminate the relationship between TG-infection and DM in males. In addition, TG-infection effects on DM could be moderated by several hidden factors that are not measured in the current study due to methodological limitations. For example, the RhD factor has been suggested as a moderator that increases the psychological effects of TG infection [85–88]. Although we have specifically addressed this variable by verifying that the same amount of Rh-positive and Rh-negative subjects were included in both experimental groups, and by controlling for RhD status in our analysis, the low prevalence of RhD-negative types in the population restricted our capability to recruit a large amount of such subjects. Furthermore, we recognize that few simple cause and effect relationships exist in nature; thus, future studies should further explore possible variables that might moderate the effects of TG on human DM, such as the time since infection, personality, levels of testosterone and cortisol, and genotype.

Finally, the current study focuses on decisions from description (that is, subjects were told about the probabilities and payoffs) rather than from experience. As people show different behavioral patterns in these two types of DM experiments [89,90], future studies should explore TG-related effects on decisions from experience. Likewise, we only investigated the link between TG infection and financial DM. Arguably, TG-infection on DM could be domain-specific (e.g., driving or health risks), or might influence decision parameters that were not explored in the current experimental setting and might be consistent with previous findings, such as under-weighting of low probabilities [91–93].

## Supporting Information

S1 Appendix Instructions. (DOCX)

S2 Appendix Parametric estimation Procedure. (DOCX)

S1 Dataset (XLSX)

S1 Fig Propensity to choose the RO as a function of its net expected utility (x-axis is truncated to (-25, 25). The figure shows the proportion of risky choices across a range of net expected utility values. The net expected utility of each binary choice (faced by each subject) was calculated by subtracting the utility of the sure option from the expected utility of the risky option. Utilities were calculated according to each subject's estimated prospect theory parameters. The choices were grouped into bins of width 5 for the purpose of calculating the proportions of risky choices. The fitted curves were obtained by estimating logit models (one model for the pooled data from each group) where the probability of choosing the risky option depends only on the net expected utility.
(PNG)

S2 Fig Propensity to choose the RO as a function of its net expected utility (x-axis is truncated to (-20, 20). The figure shows the proportion of risky choices across a range of net expected utility values. The net expected utility of each binary choice (faced by each subject) was calculated by subtracting the utility of the sure option from the expected utility of the risky option. Utilities were calculated according to each subject's estimated prospect theory parameters. The choices were grouped into bins of width 5 for the purpose of calculating the proportions of risky choices. The fitted curves were obtained by estimating logit models (one model for the pooled data from each group) where the probability of choosing the risky option depends only on the net expected utility.
(PNG)

S1 Table Summary statistics of the parametric data (full sample). (DOCX)

S2 Table Regression analysis of individual parameters (full sample). (DOCX)

S3 Table Binary choices used in the experiment as measured in CZK. (DOCX)

S4 Table Individual parameter estimates. Values in bold were excluded from the analysis.
(DOCX)

S5 Table Logistic Regression. Robustness analysis.
(DOCX)

S6 Table Mixed-effects linear regression. Robustness analysis.
(DOCX)

S7 Table Regression analysis of individual parameters. Robustness analysis.
(DOCX)
