# Supplementary material for: No Evidence of Association between Toxoplasma gondii Infection and Financial Risk Taking in Females
Source: PLoS One. 2015 Sep 24;10(9):e0136716. doi: 10.1371/journal.pone.0136716 (PMC4581702; doi:10.1371/journal.pone.0136716)
Supplement: S1 Appendix — (DOCX) [file pone.0136716.s001.docx]

**Instructions**

Introduction

Welcome to our experiment.

This is an experiment in the economics of decision making.

The instructions are simple, and if you follow them carefully and make good decisions, you might earn a considerable amount of money. Different participants may earn different amounts.

The experiment is based on simple task. In this task you will earn money. You will be paid in cash privately at the end of the experiment. Your cash payoff will be **rounded up to the next nearest 10 CZK**. Average earnings in this experiment will be **250 CZK**.

The experiment will take place through the computer terminals at which you are seated. It is important that you keep your eyes on your own screen. During the experiment, **please do not communicate with other participants**. Please turn off your mobile phone at this moment. If you have a question, please raise your hand and one of the experimenters will assist you.

**Risk Task**

Introduction

In this game you are given 250 CZK. The game consists of 140 rounds.

In each round you will choose between 50/50 gamble and sure amount. The gamble is constructed so that there is 50% chance of winning positive amount and 50% chance of losing negative amount of money (the exact amounts will be displayed on screen during each round).

At the end of 140 rounds, one round will be chosen at random for determining your payoff.

If you choose 50/50 gamble and win, positive amount of money will be added to your 250 CZK. If lose, negative amount of money will be taken from your 250 CZK. If you choose sure amount, this amount will be added to your 250CZK.

You will receive your earnings immediately after the experiment.

How to play

The task will be presented on a computer screen.

You can make your choice using a mouse click.

At the beginning of each round, 50/50 gamble and sure amount will appear. The positive amount of the gamble will be displayed in green, while the negative amount will be displayed in red. The sure amount will also be displayed in blue. If you want to choose the gamble, please click the ‘50/50’ button. If you want to choose sure amount, please click the ‘sure’ button.

After you make your choice, you will proceed to the next round.

***Important!***

Even though you do not know which one of 140 rounds will be chosen to determine your payoff, focus on each decision very carefully. Tell yourself it is the only round that matters, that this one might be the one you get paid for. As such, you might win the positive amount, but you could just as easily lose the negative amount and have to give some of 250 CZK back to the experimenter. Approach each trial as if you are making only this one choice in this game.

If you have any additional questions, please contact the experimenter, otherwise please proceed with the questions on the next page.

Thank you and good luck!

**Quiz**

**Question 1:** Your payoff in this game will be determined based on how many rounds?

**Question 2:** Imagine that, in the round, which was selected at random to calculate your payoff, you have chosen 50/50 gamble and won. The positive amount for this gamble was 79 CZK. How much will your earnings in this game be?

**Question 3:** Imagine that, in the round, which was selected at random to calculate your payoff, you have chosen 50/50 gamble and lost. The negative amount for this gamble was

-34 CZK. How much will your earnings in this game be?

**Question 4:** Imagine that, in the round, which was selected at random to calculate your payoff, you have chosen sure amount and it was 22 CZK. How much will your earnings in this game be?
