# Supplementary material for: No Evidence of Association between Toxoplasma gondii Infection and Financial Risk Taking in Females
Source: PLoS One. 2015 Sep 24;10(9):e0136716. doi: 10.1371/journal.pone.0136716 (PMC4581702; doi:10.1371/journal.pone.0136716)
Supplement: S2 Appendix — (DOCX) [file pone.0136716.s002.docx]

**Parametric estimation Procedure**

We conducted a parametric analysis to estimate risk aversion and loss-aversion via a nonlinear stochastic choice model. Following Tversky and Kahneman [83], we represent subject’s utility functions for money as a two-part power function of the form

$$u(x)=\left\{\begin{matrix} x^{\rho} for all x\geq0 \\ -\lambda(-x)^{\rho} for all x<0 \end{matrix}\right.$$

The loss aversion coefficient λ represents relative multiplicative weighting of losses compared to gains. The function’s exponential form captures the empirical regularity of risk aversion [56-57], such that the parameter ρ represents diminishing sensitivity to changes in monetary value as its absolute amount increases. Monetary outcomes are raised to a power equal to their value -- producing an exponential curve which is concave for gains and convex for losses (if ρ <1). A smaller value of ρ represents a higher rate of diminishing sensitivity and more risk aversion, relative to a larger ρ, where ρ=1 implies no diminishing sensitivity, i.e., risk neutrality. The diminishing sensitivity represented by ρ is equivalent to risk aversion in the gain domain and risk seeking in the loss domain, as demonstrated by the following example. Consider a gamble of $20/$0 compared to a guaranteed amount of $10. The objective expected value of the gamble is $10 (expected value = probability x value, or 0.5 x $20 + 0.5 x $0 = $10), equal to the guaranteed amount. Therefore, a risk neutral individual would be indifferent between this gamble and the guaranteed amount. However, because the subjective value equation is exponential, the $20 in the gamble is discounted relatively more than the $10 in the guaranteed amount, thus leaving the gamble with a lower subjective value and leading the individual to reject the gamble for the guaranteed amount (risk averse behavior). As an example, if ρ=0.87 (the average value in our data), the gamble would have a subjective value of 6.77, and the guaranteed amount a subjective value of 7.52. The degree of curvature of the utility function is identical for the gain and the loss domains [56-57]. We further assume that people combine probabilities and utilities linearly, in the form . As in our experiment *p* equals 0.5 for all the risky prospects; thus, nonlinear weighting of probabilities [56-57, 83] applies equally to all choices, leaving our results qualitatively unchanged.[[1]](#footnote-1)

The probability that a subject chooses the uncertain prospect rather than the degenerate prospect is given by the logit function

$$F(p,G,L,SO)=\frac{1}{1+\exp\{-\mu[U(p,G,L)-U(SO)]\}},$$

where *G* and *L* are the positive and negative outcomes of the risky prospects respectively, and *SO* the outcome of the certain prospect. The logit parameter, *μ,* is the sensitivity of choice probability to the utility difference, or the amount of ‘randomness’ in the subject’s choices (*μ* equals 0 means choices are random; as *μ* increases the function is more steeply inflected at zero).

We fit the data using maximum likelihood, by maximizing the log likelihood function:

1. $\sum_{i=1}^{140} y_{i}\log(F(p,G,L,SO))+(1-y_{i})\log(1-F(p,G,L,SO))$,

such that$y_{i}$equals 1 for choices of the risky option and 0 for choices of the safe option. To solve this nonlinear optimization problem, we used a grid search optimization algorithm implemented in MATLAB, with resolution of 0.01 for all parameter values, and a greed search areas that fully contained the parameter values intervals found in previous studies using the same task:$\rho\in[0.2 .. 2]$,$\lambda\in[0.3 .. 4]$ , $\mu\in[0.2-2]$ [56-57].

1. Various studies reported that the magnitude of underweighting at *p=0.5* is small, with an estimated weight of *w(0.5) = 0.45* [84] [↑](#footnote-ref-1)
