# Supplementary figures and images for: No Evidence of Association between Toxoplasma gondii Infection and Financial Risk Taking in Females
Source: PLoS One. 2015 Sep 24;10(9):e0136716. doi: 10.1371/journal.pone.0136716 (PMC4581702; doi:10.1371/journal.pone.0136716)

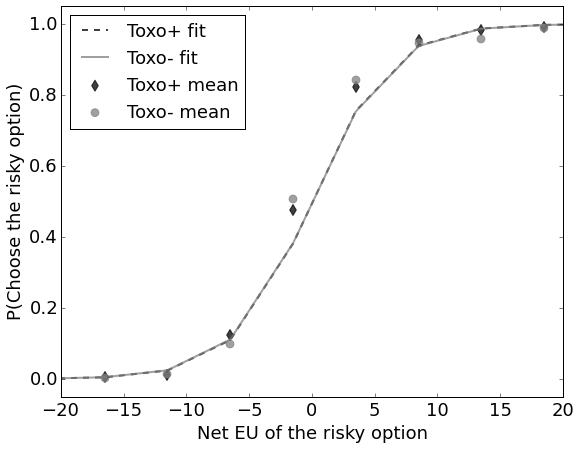

Supplement: S1 Fig — The figure shows the proportion of risky choices across a range of net expected utility values. The net expected utility of each binary choice (faced by each subject) was calculated by subtracting the utility of the sure option from the expected utility of the risky option. Utilities were calculated according to each subject's estimated prospect theory parameters. The choices were grouped into bins of width 5 for the purpose of calculating the proportions of risky choices. The fitted curves were obtained by estimating logit models (one model for the pooled data from each group) where the probability of choosing the risky option depends only on the net expected utility. (PNG) [file pone.0136716.s004.png]

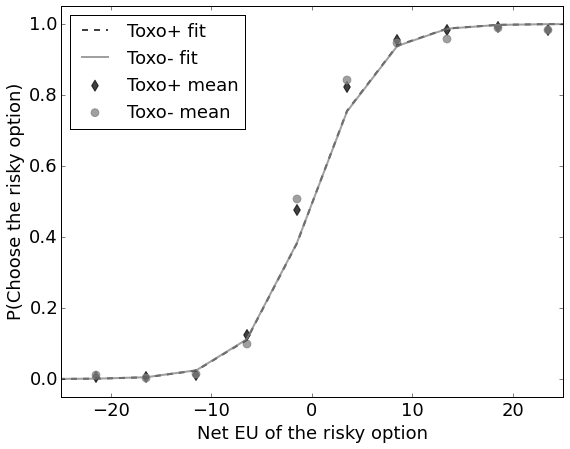

Supplement: S2 Fig — The figure shows the proportion of risky choices across a range of net expected utility values. The net expected utility of each binary choice (faced by each subject) was calculated by subtracting the utility of the sure option from the expected utility of the risky option. Utilities were calculated according to each subject's estimated prospect theory parameters. The choices were grouped into bins of width 5 for the purpose of calculating the proportions of risky choices. The fitted curves were obtained by estimating logit models (one model for the pooled data from each group) where the probability of choosing the risky option depends only on the net expected utility. (PNG) [file pone.0136716.s005.png]
