# Supplementary material for: No Evidence of Association between Toxoplasma gondii Infection and Financial Risk Taking in Females
Source: PLoS One. 2015 Sep 24;10(9):e0136716. doi: 10.1371/journal.pone.0136716 (PMC4581702; doi:10.1371/journal.pone.0136716)
Supplement: S1 Table — (DOCX) [file pone.0136716.s006.docx]

Table S1. Summary statistics of the parametric data (sample with 70 subjects).

| ^All Toxo + Toxo - P^  ^Variable Obs Mean Std. Dev. Obs Mean Std. Dev. Obs Mean Std. Dev.^ |
| --- |
| ^ρ 70 0.877 0.097 32 0.88 0.109 38 0.875 0.088 0.833^  ^λ 70 1.409 0.553 32 1.415 0.571 38 1.404 0.546 0.932^ |

Notes: P shows statistical significance for two tailed t-test.
