# Supplementary material for: No Evidence of Association between Toxoplasma gondii Infection and Financial Risk Taking in Females
Source: PLoS One. 2015 Sep 24;10(9):e0136716. doi: 10.1371/journal.pone.0136716 (PMC4581702; doi:10.1371/journal.pone.0136716)
Supplement: S2 Table — (DOCX) [file pone.0136716.s007.docx]

Table S2. Regression analysis of individual parameters (sample with 70 subjects).

| ^ρ λ^  ^Dependent Variable (1) (3)^ |
| --- |
| *^Toxoplasma^* ^0.040 -0.099^  ^(0.042) (0.242)^  ^Age -0.0006 0.018^  ^(0.003) (0.018).^  ^RhD 0.041 0.043^  ^(0.034) (0.196)^  *^Toxoplasma^*^*RhD -0.049 0.120^  ^(0.051) (0.291)^  ^Constant 0.861*** 0.936**^  ^(0.079) (0.447)^ |

R^2^ 0.023 0.027

Observations 70 70

Notes: *Toxoplasma* is a dummy variable and equals 1 for *Toxoplasma*-infected subjects. RhD is a dummy variable and equals 1 for RhD positive subjects. Coefficients in all columns OLS regression estimates, standard errors are in parentheses; ***, **, and * indicate significance at 1%, 5%, and 10% level, respectively.
