# Supplementary material for: No Evidence of Association between Toxoplasma gondii Infection and Financial Risk Taking in Females
Source: PLoS One. 2015 Sep 24;10(9):e0136716. doi: 10.1371/journal.pone.0136716 (PMC4581702; doi:10.1371/journal.pone.0136716)
Supplement: S3 Table — (DOCX) [file pone.0136716.s008.docx]

**Table S3.** Binary choices used in the experiment as measured in CZK.

**Gain Loss SO Gain Loss SO Gain Loss SO**

| 38 CZK 0 CZK 19 CZK  95 CZK 119 CZK 0 CZK  48 CZK 18 CZK 0 CZK  48 CZK 65 CZK 0 CZK  38 CZK 38 CZK 0 CZK  48 CZK 30 CZK 0 CZK  95 CZK 36 CZK 0 CZK  95 CZK 24 CZK 0 CZK  114 CZK 214 CZK 0 CZK  114 CZK 114 CZK 0 CZK  114 CZK 0 CZK 57 CZK  48 CZK 36 CZK 0 CZK  19 CZK 17 CZK 0 CZK  19 CZK 0 CZK 10 CZK  57 CZK 78 CZK 0 CZK  19 CZK 10 CZK 0 CZK  95 CZK 83 CZK 0 CZK  238 CZK 0 CZK 95 CZK  76 CZK 86 CZK 0 CZK  38 CZK 10 CZK 0 CZK  285 CZK 0 CZK 114 CZK  238 CZK 0 CZK 86 CZK  76 CZK 95 CZK 0 CZK  114 CZK 157 CZK 0 CZK  19 CZK 29 CZK 0 CZK  95 CZK 143 CZK 0 CZK  57 CZK 71 CZK 0 CZK  95 CZK 190 CZK 0 CZK  38 CZK 76 CZK 0 CZK  209 CZK 0 CZK 95 CZK  48 CZK 95 CZK 0 CZK  86 CZK 32 CZK 0 CZK  57 CZK 114 CZK 0 CZK  114 CZK 128 CZK 0 CZK  76 CZK 143 CZK 0 CZK  86 CZK 43 CZK 0 CZK  76 CZK 124 CZK 0 CZK  76 CZK 29 CZK 0 CZK  76 CZK 105 CZK 0 CZK  114 CZK 0 CZK 38 CZK  114 CZK 43 CZK 0 CZK  219 CZK 0 CZK 95 CZK  57 CZK 50 CZK 0 CZK  48 CZK 24 CZK 0 CZK  19 CZK 19 CZK 0 CZK  86 CZK 128 CZK 0 CZK  95 CZK 166 CZK 0 CZK |  | 48 CZK 53 CZK 0 CZK  95 CZK 131 CZK 0 CZK  57 CZK 100 CZK 0 CZK  86 CZK 21 CZK 0 CZK  86 CZK 75 CZK 0 CZK  86 CZK 160 CZK 0 CZK  48 CZK 77 CZK 0 CZK  38 CZK 67 CZK 0 CZK  114 CZK 0 CZK 48 CZK  38 CZK 14 CZK 0 CZK  124 CZK 0 CZK 57 CZK  48 CZK 71 CZK 0 CZK  114 CZK 228 CZK 0 CZK  57 CZK 21 CZK 0 CZK  114 CZK 100 CZK 0 CZK  95 CZK 107 CZK 0 CZK  86 CZK 64 CZK 0 CZK  19 CZK 36 CZK 0 CZK  57 CZK 14 CZK 0 CZK  57 CZK 86 CZK 0 CZK  76 CZK 38 CZK 0 CZK  95 CZK 178 CZK 0 CZK  95 CZK 48 CZK 0 CZK  114 CZK 185 CZK 0 CZK  19 CZK 21 CZK 0 CZK  76 CZK 76 CZK 0 CZK  86 CZK 107 CZK 0 CZK  266 CZK 0 CZK 124 CZK  86 CZK 171 CZK 0 CZK  95 CZK 95 CZK 0 CZK  247 CZK 0 CZK 114 CZK  86 CZK 96 CZK 0 CZK  57 CZK 107 CZK 0 CZK  114 CZK 29 CZK 0 CZK  86 CZK 53 CZK 0 CZK  19 CZK 14 CZK 0 CZK  57 CZK 29 CZK 0 CZK  114 CZK 57 CZK 0 CZK  57 CZK 64 CZK 0 CZK  38 CZK 71 CZK 0 CZK  38 CZK 33 CZK 0 CZK  38 CZK 52 CZK 0 CZK  57 CZK 57 CZK 0 CZK  114 CZK 71 CZK 0 CZK  48 CZK 48 CZK 0 CZK  76 CZK 48 CZK 0 CZK  48 CZK 12 CZK 0 CZK |  | 57 CZK 36 CZK 0 CZK  38 CZK 62 CZK 0 CZK  114 CZK 86 CZK 0 CZK  76 CZK 19 CZK 0 CZK  86 CZK 150 CZK 0 CZK  57 CZK 43 CZK 0 CZK  86 CZK 139 CZK 0 CZK  38 CZK 57 CZK 0 CZK  48 CZK 83 CZK 0 CZK  48 CZK 0 CZK 19 CZK  95 CZK 59 CZK 0 CZK  38 CZK 24 CZK 0 CZK  95 CZK 154 CZK 0 CZK  48 CZK 89 CZK 0 CZK  124 CZK 0 CZK 48 CZK  19 CZK 38 CZK 0 CZK  76 CZK 133 CZK 0 CZK  19 CZK 5 CZK 0 CZK  67 CZK 0 CZK 29 CZK  48 CZK 42 CZK 0 CZK  114 CZK 171 CZK 0 CZK  86 CZK 86 CZK 0 CZK  76 CZK 114 CZK 0 CZK  38 CZK 43 CZK 0 CZK  76 CZK 0 CZK 29 CZK  247 CZK 0 CZK 95 CZK  76 CZK 57 CZK 0 CZK  19 CZK 7 CZK 0 CZK  181 CZK 0 CZK 76 CZK  114 CZK 143 CZK 0 CZK  19 CZK 12 CZK 0 CZK  38 CZK 19 CZK 0 CZK  86 CZK 118 CZK 0 CZK  76 CZK 152 CZK 0 CZK  38 CZK 48 CZK 0 CZK  95 CZK 71 CZK 0 CZK  48 CZK 59 CZK 0 CZK  19 CZK 33 CZK 0 CZK  19 CZK 24 CZK 0 CZK  76 CZK 67 CZK 0 CZK  38 CZK 29 CZK 0 CZK  57 CZK 93 CZK 0 CZK  29 CZK 0 CZK 10 CZK  19 CZK 26 CZK 0 CZK  19 CZK 31 CZK 0 CZK  114 CZK 200 CZK 0 CZK |
| --- | --- | --- | --- | --- |
