# Supplementary material for: No Evidence of Association between Toxoplasma gondii Infection and Financial Risk Taking in Females
Source: PLoS One. 2015 Sep 24;10(9):e0136716. doi: 10.1371/journal.pone.0136716 (PMC4581702; doi:10.1371/journal.pone.0136716)
Supplement: S4 Table — Values in bold were excluded from the analysis. (DOCX) [file pone.0136716.s009.docx]

**Table S4.** Individual parameter estimates. Values in bold were excluded from the analysis.

**ID ρ λ TG Status ID ρ λ TG Status**

| 1  2  3  4  5  6  7  8  9  10  11  12  13  14  15  16  17  18  19  20  21  22  23  24  25  26  27  28  29  30  31  32  33  34  35  36  37  38  39  40 | 0.93 1.18 0  0.84 2.03 1  **0.71 4 1**  0.67 1.8 1  0.9 1.25 1  0.78 0.76 0  **0.82 3.67 1**  0.91 0.89 1  1.05 1.21 0  0.91 0.74 1  **0.85 4 0**  1.07 1.29 1  0.86 2.28 1  1.11 0.95 1  0.61 3.25 1  0.84 1.17 0  0.75 1.71 1  0.84 1.49 1  0.97 1.41 1  0.79 1.62 0  0.73 1.31 1  0.85 1.23 0  0.74 1.61 0  **0.5 4 1**  0.87 1.54 0  0.86 1.11 1  0.77 1.42 1  0.89 1.28 1  **0.2 4 1**  0.89 1.76 1  0.98 1.78 0  0.81 1.38 0  0.95 1.58 0  0.93 1.12 0  0.9 0.78 1  0.97 0.94 0  1 0.94 0  0.92 1.29 0  0.81 1.75 0  0.9 2.41 1 | 41  42  43  44  45  46  47  48  49  50  51  52  53  54  55  56  57  58  59  60  61  62  63  64  65  66  67  68  69  70  71  72  73  74  75  76  77  78  79 | 0.99 2.99 0  0.92 1.18 0  0.86 1.29 0  0.77 0.94 0  0.85 3.3 0  0.88 1.09 1  0.81 0.74 0  **0.6 3.48 1**  0.92 1.22 0  0.86 1.71 0  0.82 1.14 1  1.02 1.67 1  0.92 1.01 1  0.89 1.34 0  0.88 1.45 0  0.97 1.52 0  0.84 2.22 1  1.05 1.86 1  0.81 1.1 0  0.79 1.73 0  0.95 0.56 1  **0.46 0.3 1**  0.91 0.89 1  0.92 1.37 1  0.94 1.18 1  0.71 0.88 1  0.93 1.41 0  0.83 1.24 0  0.98 1.09 0  **0.89 4 1**  0.88 1.13 1  0.83 2.43 0  0.59 0.74 0  0.92 0.65 0  **0.46 1.26 0**  0.92 1.08 0  0.94 1.14 1  0.9 1.67 0  0.81 1.44 0 |
| --- | --- | --- | --- |
