# Supplementary material for: No Evidence of Association between Toxoplasma gondii Infection and Financial Risk Taking in Females
Source: PLoS One. 2015 Sep 24;10(9):e0136716. doi: 10.1371/journal.pone.0136716 (PMC4581702; doi:10.1371/journal.pone.0136716)
Supplement: S5 Table — Robustness analysis. (DOCX) [file pone.0136716.s010.docx]

Table S5. Logistic regression (Robustness analysis).

| ^Choice^  ^Dependent Variable (1) (2) (3) (4)^ |
| --- |
| *^Toxoplasma^* ^-0.171 0.083 -0.172 0.084^  ^(0.258) (0.477) (0.260) (0.482)^  ^Incentive 0.086*** 0.086*** 0.086*** 0.086***^  ^(0.007) (0.007) (0.008) (0.008)^  ^Age 0.0005 0.0005^  ^(0.032) (0.033)^  ^RhD -0.029 -0.029^  ^(0.358) (0.362)^  *^Toxoplasma^*^*RhD -0.387 -0.389^  ^(0.562) (0.567)^  ^Variance -6.04E-5*** -6.06E-5***^  ^(1.52E-5) (1.51E-5)^  ^Constant -0.511*** -0.505 -0.231 -0.224^  ^(0.168) (0.800) (0.170) (0.809)^ |

Restricted Log Likelihood -5024 -5007 -4960 -4943

Observations 10220 10220 10220 10220

Notes: Choice is a dummy variable and equals 1 if subjects chose risky option. *Toxoplasma* is a dummy variable and equals 1 for *Toxoplasma*-infected subjects. RhD is a dummy variable and equals 1 for RhD positive subjects. Coefficients in all columns are logistic regression estimates, clustered standard errors are in parentheses; ***, **, and * indicate significance at 1%, 5%, and 10% level, respectively.
