# Supplementary material for: No Evidence of Association between Toxoplasma gondii Infection and Financial Risk Taking in Females
Source: PLoS One. 2015 Sep 24;10(9):e0136716. doi: 10.1371/journal.pone.0136716 (PMC4581702; doi:10.1371/journal.pone.0136716)
Supplement: S6 Table — Robustness analysis. (DOCX) [file pone.0136716.s011.docx]

Table 3. Mixed-effects linear regression (Robustness analysis).

| ^Response Time^  ^Dependent Variable (1) (2) (3)^ |
| --- |
| *^Toxoplasma^* ^0.259 0.190 -0.088^  ^(0.263) (0.274) (0.470)^  ^Incentive 0.011*** 0.021*** 0.021***^  ^(0.001) (0.002) (0.002)^  ^Choice 0.793*** 0.975*** 0.976***^  ^(0.078) (0.106) (0.106)^  ^Toxoplasma*Choice 0.131 0.131^  ^(0.129) (0.129)^  ^Choice*Incentive -0.039*** -0.039***^  ^(0.004) (0.004)^  ^Age 0.040^  ^(0.036)^  ^RHD 0.183^  ^(0.412)^ *^Toxoplasma^*^*RHD 0.309^  ^(0.562)^  ^Constant 3.150*** 3.280*** 2.230**^  ^(0.195) (0.201) (0.880)^ |

Log Likelihood -25733 -25687 -25685

Observations 10220 10220 10220

Notes: Choice is a dummy variable and equals 1 if subjects chose risky option. *Toxoplasma* is a dummy variable and equals 1 for *Toxoplasma*-infected subjects. RhD is a dummy variable and equals 1 for RhD positive subjects. Coefficients in all columns are mixed effects linear regression estimates, standard errors are in parentheses; ***, **, and * indicate significance at 1%, 5%, and 10% level, respectively.
