# Supplementary material for: No Evidence of Association between Toxoplasma gondii Infection and Financial Risk Taking in Females
Source: PLoS One. 2015 Sep 24;10(9):e0136716. doi: 10.1371/journal.pone.0136716 (PMC4581702; doi:10.1371/journal.pone.0136716)
Supplement: S7 Table — Robustness analysis. (DOCX) [file pone.0136716.s012.docx]

Table 5. Regression analysis of individual parameters (Robustness analysis).

| ^ρ λ^  ^Dependent Variable (1) (3)^ |
| --- |
| *^Toxoplasma^* ^0.048 -0.041^  ^(0.048) (0.262)^  ^Age -0.0007 0.019^  ^(0.003) (0.019).^  ^RhD 0.052 0.140^  ^(0.038) (0.222)^  *^Toxoplasma^*^*RhD -0.060 0.023^  ^(0.054) (0.313)^  ^Constant 0.855*** 0.873*^  ^(0.081) (0.466)^ |

R^2^ 0.032 0.034

Observations 64 64

Notes: *Toxoplasma* is a dummy variable and equals 1 for *Toxoplasma*-infected subjects. RhD is a dummy variable and equals 1 for RhD positive subjects. Coefficients in all columns OLS regression estimates, standard errors are in parentheses; ***, **, and * indicate significance at 1%, 5%, and 10% level, respectively.
